# Real-World Management of HMA-Related Myelosuppression During MDS Treatment in the Canadian Landscape

**DOI:** 10.3390/curroncol33070404

**Published:** 2026-07-07

**Authors:** Michelle Geddes, Brett L. Houston, Lalit Saini, Ismail Sharif, Rena Buckstein, Ryan J. Stubbins

**Affiliations:** 1 Division of Hematology and Hematologic Malignancies, Arthur J.E. Child Comprehensive Cancer Centre, Calgary, AB T2N 5G2, Canada; michelle.geddes@albertahealthservices.ca; 2 Max Rady Faculty of Health Sciences, University of Manitoba, Winnipeg, MB R3E 0W3, Canada; bhouston@cancercare.mb.ca; 3 CancerCare Manitoba, Winnipeg, MB R3E 0V9, Canada; 4 London Health Sciences Centre, London, ON N6A 5W9, Canada; lalit.saini@lhsc.on.ca; 5 Department of Medicine, The University of Western Ontario, London, ON N6A 3K7, Canada; 6 Peterborough Regional Health Centre, Peterborough, ON K9J 7C6, Canada; issharif@lh.ca; 7 Odette Cancer Centre, Sunnybrook Health Sciences Centre, Toronto, ON M4N 3M5, Canada; rena.buckstein@sunnybrook.ca; 8 Leukemia/BMT Program of British Columbia, Vancouver Coastal Health, BC Cancer, Vancouver, BC V5Z 1M9, Canada

**Keywords:** myelodysplastic syndromes, hypomethylating agents, azacitidine, decitabine-cedazuridine, myelosuppression, supportive care

## Abstract

Hypomethylating agents (HMAs) are the cornerstone in the treatment of higher-risk myelodysplastic syndromes (MDSs), particularly for patients who are not candidates for allogeneic hematopoietic cell transplant (allo-HCT). Despite demonstrated efficacy in improving hematologic outcomes, the clinical management of HMA-associated myelosuppression remains a challenge. This review discusses the use of azacitidine and oral decitabine-cedazuridine (DEC-C) for MDS management in the Canadian context, with a focus on optimizing therapy to mitigate myelosuppression and prevent early HMA discontinuation due to toxicity. Close monitoring of complete blood counts is critical to early detection of myelosuppression and management of treatment-related cytopenias. In the real-world setting, specific HMA dose adjustments are used based on patient risk factors for myelosuppression or treatment-related complications. Supportive care strategies, including the use of growth factors and antimicrobials, can complement monitoring and dose modifications for HMA-related myelosuppression management, although their use is variable. This review summarizes current evidence and real-world management approaches for HMA-induced myelosuppression, with the aim of improving outcomes for patients undergoing treatment for MDS.

## Simple Summary

Hypomethylating agents (HMAs), azacitidine and oral decitabine-cedazuridine, are treatments for myelodysplastic syndrome (MDS), a type of blood cancer. While these treatments can be effective in targeting the cancer, they often damage the body’s ability to create blood cells in the bone marrow, leading to low levels of blood cells (particularly white blood cells, red blood cells, and the cells which clot blood) as a side effect known as myelosuppression. This side effect may lead to treatment being stopped due to risk of infection or bleeding because of low blood cell levels, preventing patients from benefiting from the hypomethylating agent’s positive effects in treating the cancer. This review summarizes strategies for managing HMA-related myelosuppression in Canadian patients with MDS. These strategies include close monitoring of blood cell levels, adjusting HMA dose based on patient needs, and other interventions to boost blood cells and/or protect against infection. By managing these side effects, physicians can help patients continue their treatment longer, thereby improving their chances of better outcomes.

## 1. Introduction

### 1.1. Current Canadian Landscape of HMA Treatment for MDS

Hypomethylating agents (HMAs) remain the cornerstone for the management of myelodysplastic syndromes (MDS) and are considered standard of care, particularly for patients with higher-risk disease [1]. This review is based on the current Canadian regulatory and clinical landscape for HMA use in MDS, where two HMAs, parenteral (subcutaneous, intravenous) azacitidine and oral decitabine-cedazuridine (DEC-C), are approved and available for the treatment of MDS [2,3,4,5]. Intravenous decitabine is also used for MDS treatment [6,7,8], but access and availability vary. It should be noted that oral DEC-C is not currently approved or available for treating MDS in some countries. Within Canada, approval specifics of azacitidine and oral DEC-C vary by jurisdiction, but the Molecular International Prognostic Scoring System (IPSS-M) is largely used by clinicians for risk stratification and to determine eligibility for HMA treatment in the current era. The International Prognostic Scoring System (IPSS), International Prognostic Scoring System-Revised (IPSS-R), and IPSS-M risk stratification systems are summarized in Table 1. Patients are typically considered for HMA treatment if they have higher-risk disease, defined as an IPSS-M score that is considered moderate-high, high, or very high risk [1,9]. Some patients with low-risk disease and symptomatic cytopenias may also benefit from HMAs, such as those with transfusion-dependent anemia who have failed or are ineligible for erythropoiesis-stimulating agents [10,11].

Unlike allogeneic hematopoietic cell transplant (allo-HCT), treatment of MDS with single-agent HMA is palliative rather than curative, although HMA is often used as a bridge to allo-HCT in eligible patients with excess blasts or fast kinetic disease [14,15]. However, single-agent HMAs have been associated with hematologic improvements, enhanced quality of life, transfusion independence, and delayed progression to acute myeloid leukemia (AML) [2,4,6]. Amongst patients with higher-risk MDS, azacitidine demonstrated a survival advantage over conventional care in the AZA-001 trial (higher-risk MDS defined in this article as IPSS intermediate-2 [INT-2] or high risk) and in systematic review [2,16]. Despite these benefits, MDS outcomes remain poor, highlighting the need for improvement in MDS management [17,18].

### 1.2. Review of Efficacy and Safety of Currently Available HMAs

Evidence shows that HMAs are frequently underused or sub-optimally used [19,20,21,22,23,24]. Patients with higher-risk MDS (refractory anemia with excess blasts [RAEB] designation as proxy in this article) who are not receiving HMAs experienced greater healthcare resource utilization, including more monthly hospitalizations and emergency room (ER) visits, compared with those receiving HMAs [23]. These findings underscore the importance of improving HMA utilization in the management of MDS.

Patients receiving HMAs typically achieve their best response at 4–6 months, although improvements can be seen as early as 2 months (Table 2) [15,25]. Despite these benefits, only 40–50% of patients with MDS complete the first 4 cycles of HMA treatment, and only 30–40% complete 6 cycles, depending on the HMA used [19]. Amongst patients who receive 6 or more HMA cycles, survival outcomes are known to be improved compared with those with early discontinuation (i.e., <6 cycles) [26,27]. Early discontinuation is often due to lack of response, progressive disease, or the occurrence of toxicity. Cytopenias are a significant risk factor for early treatment discontinuation [19], highlighting the need for optimal management during the early cycles to maximize the chance of achieving best response.

### 1.3. Myelosuppression with HMA Use

Myelosuppression is the most common side effect associated with HMAs (Table 3) and is of particular clinical relevance given the vulnerability of the patient population [29]. Up to 25% of patients with MDS are considered frail [30], ∼83% are ≥65 years in age, and patients often present with multiple comorbidities at diagnosis [31,32]. These features make this group particularly at risk of complications related to cytopenias, such as bleeding or infection, which likely contributes to the lower use of HMAs in older and frail patient subgroups [19].

Rates of treatment discontinuation due to adverse events (AEs) in the clinical trials were generally low (2% for oral DEC-C and 5% due to hematologic AEs for azacitidine [2,4]), whereas real-world discontinuation rates due to AEs were higher, ranging from 3.4–8.7% for oral DEC-C and 14.4–18.5% for azacitidine [27,33,34]. Discontinuation rates due to toxicities were even higher for combination therapy: ~20% for azacitidine + lenalidomide or vorinostat, with ~40% of patients undergoing nonprotocol-defined dose modifications [35].

The focus of this review is to summarize the current evidence and real-world approaches to managing myelosuppression during HMA treatment, with the goal of optimizing the use of HMAs in practice and improving outcomes for patients with MDS. The practices described here are based on a combination of evidence from randomized Phase III trials, prospective and retrospective data where available (albeit data is limited), as well as the practical clinical experience of the authors, and may fall outside the scope of the approved Canadian product monographs for azacitidine and oral DEC-C. Practices described herein were classified as evidence-based when supported by formally published guidelines or recommendations from recognized health agencies, including Health Canada and the FDA. Where such evidence was not available, practices were classified as expert opinion if shared agreement was reached among all authors through virtual meetings and asynchronous discussion.

## 2. HMA Initiation

### 2.1. Choice of HMA

HMAs are considered the first-line option for all patients with higher-risk MDS (defined by a score of moderate-high, high, or very high risk per IPSS-M) who are not transplant-eligible [15], and can be considered as a bridge to transplant in patients who are potentially eligible for allo-HCT [15,36]. Assessment is made regarding comorbidities and frailty to help evaluate whether the patient is fit for HMA therapy [30,37]. Factors that may make a patient unfit for HMA therapy include, but are not limited to, severe organ system dysfunction, active uncontrolled infection, uncontrolled or life-threatening systemic diseases, lab abnormalities that could compromise patient safety on treatment, or poor patient performance status (expert opinion). Predictive biomarkers (e.g., *TP53*, *TET2* mutations [38,39]) are an area of interest to potentially optimize HMA response, but data remain limited, and thus they are not currently used for selection of patients with MDS suitable for HMA treatment (expert opinion).

The choice between azacitidine and oral DEC-C for treating MDS often depends on factors such as patient’s proximity to the treatment center, patient preference, and jurisdictional drug funding (expert opinion). Considering subcutaneous versus oral HMA therapy should involve shared decision making that accounts for patient preferences and potential impact on treatment adherence (expert opinion). Oral HMA therapy may often be preferred by patients because of its convenience and potential to reduce the need for healthcare visits or interactions. Accordingly, oral DEC-C may help reduce treatment burden compared with parenteral HMA therapy, evidenced by little interference with daily activities and improved quality of life relative to the injectable treatments [40].

Clinicians also consider contraindications and drug–drug interactions when choosing an HMA (expert opinion). Azacitidine is contraindicated in patients with advanced malignant hepatic tumors [3] (evidence-based). Azacitidine and oral DEC-C are not metabolized by cytochrome P450 (CYP450) enzymes, and thus interactions with CYP inhibitors/inducers are unlikely. Oral DEC-C co-administration should be avoided with drugs metabolized by cytidine deaminase (e.g., cytarabine, gemcitabine, azacitidine, and some antivirals) [3,5] (evidence-based). Oral DEC-C is not expected to affect P-glycoprotein (P-gp)-mediated transport of co-administered medications (evidence-based). Based on a population pharmacokinetic (PK) analysis, no effect on cedazuridine or decitabine PK was shown with gastric pH-modifying drugs as long as they are not administered within 4 h of oral DEC-C administration [5] (evidence-based). Oral DEC-C should not be taken with food. Based on limited data, taking oral DEC-C with a meal could reduce overall decitabine exposure [5], and it is best taken on an empty stomach (evidence-based).

### 2.2. Dosing Considerations

The starting dose of azacitidine for the first treatment cycle is 75 mg/m^2^ of body surface area, injected subcutaneously daily for 7 consecutive days followed by a 21-day rest period (28-day treatment cycle) as per Health Canada, U.S. Food and Drug Administration (FDA), and European Medicines Agency (EMA) [3,41,42]. The starting dose for azacitidine is based on the AZA-001 clinical trial, which included younger, less frail patients (i.e., median age 69 years, mostly with Eastern Cooperative Oncology Group [ECOG] score 0 or 1), compared with those with higher-risk MDS typically seen in clinical practice [2]. The health agency-recommended starting dose of azacitidine is typically used in real-world practice [27,43], as complications of severe myelosuppression are less common with azacitidine (expert opinion). Initially, challenges were encountered in managing azacitidine-related myelosuppression, but with increased experience, clinicians have become more comfortable managing less severe cytopenias. An alternative dosing regimen (i.e., 5-2-2), with daily administration on days 1–5 and 8–9, is effective and may be used to accommodate clinic hours, with many treatment centers having limited weekend availability [44,45,46,47,48] (expert opinion).

Oral DEC-C is administered as a fixed-dose single tablet. The health agency-recommended starting dose is 1 tablet (35 mg of decitabine and 100 mg of cedazuridine) once daily on days 1 through 5 of each 28-day cycle as per Health Canada, FDA and EMA [5,49,50]. Since oral DEC-C is available as a fixed-dose tablet, modification involves changing the frequency at which it is administered. The approaches discussed here reflect reported real-world practice and are supported by limited evidence, with most decisions relying on clinician experience.

In real-world practice, clinicians assess a patient’s risk of myelosuppression and treatment-related complications to determine whether a reduced starting dose frequency of oral DEC-C may be appropriate, with the goal of completing a minimum of 4–6 cycles of treatment to evaluate response. In patients at higher risk of myelosuppression (e.g., advanced age, frail individuals, low body weight, severe baseline pancytopenia, bone marrow fibrosis, hypoplastic MDS, germline predisposition syndromes), as well as those at risk for treatment-related complications (e.g., falls, hospitalizations, poor performance status, bleeding, or infectious complications), dose reduction to once daily dosing on days 1–3 or 1–4 of the cycle may be considered as a starting regimen of oral DEC-C (expert opinion). This is reflective of real-world experience, where 35% of patients received modified cycles at the initiation of treatment with oral DEC-C [27]. In a Phase I/II study of oral DEC-C in patients with lower-risk MDS, a 3-day dosing regimen showed clinical efficacy in terms of hematologic improvement and transfusion independence, but this has not been studied in a Phase III trial [51]. For patients with liver disease or renal failure, even lower starting dose frequency may be considered, such as a 3-day regimen administered every other day (i.e., days 1, 3, 5), 2-day regimen (i.e., days 1–2), or 1-day regimen (i.e., day 1) (expert opinion). In a retrospective real-world Canadian cohort of 50 patients with MDS (*n* = 44) or chronic myelomonocytic leukemia (CMML) (*n* = 6), overall survival (OS) was better in the shortened oral DEC-C cohort who received a 3-day regimen compared with the 5-day regimen (not reached vs. 13 months; *p* = 0.04. Patients receiving an HMA with ≥25% of cycles modified or delayed also had longer OS without a detrimental impact on PFS compared with those with <25% of cycles modified/delayed [27]. 

In clinical practice, when patients begin treatment with a reduced dose of oral DEC-C, the dose is rarely increased later due to concerns of toxicity arising, which could prevent completion of the minimum 4–6 treatment cycles. However, if a patient tolerates the first cycle well, a gradual escalation to the health agency-recommended starting dose may be considered before achievement of hematologic improvement, especially if their complete blood counts are nearer to baseline (expert opinion).

The standard 5-day starting dose of oral DEC-C is more commonly used for patients at lower risk of myelosuppression and related complications, such as those <65 years of age with no significant comorbidities, patients with high starting blast counts, or those with proliferative CMML, who experience less myelosuppression with oral DEC-C than those with MDS, based on anecdotal evidence (expert opinion). Current prospective data suggest the real-world safety profile for oral DEC-C in patients with CMML is comparable to what was observed in the Phase II and III studies for MDS and CMML [52]. No differences in clinical outcomes (OS or event-free survival [EFS]) were observed with decitabine vs. hydroxyurea in patients with proliferative CMML in the DACOTA trial [53]; however, recent data suggest favorable outcomes with use of HMAs in these patients and warrant further study [52,54,55].

### 2.3. Dosing Considerations for Patients Who Are Transplant-Eligible

Although HMAs are primarily used for patients with MDS who are ineligible for transplant, they may be used to reduce disease burden or prevent disease progression prior to transplant. Large, retrospective studies have demonstrated higher blast counts at transplantation and higher risk scores (as determined by IPSS) are associated with higher risk of relapse in patients with MDS and CMML [56,57], although the benefit of cytoreduction from retrospective transplant literature is unproven [58]. Given this, the current recommendation of an international expert panel of bone marrow transplant societies is to perform cytoreduction with induction chemotherapy or HMA prior to allo-HCT in high-risk patients with MDS with ≥10% marrow blasts [59] (evidence-based). In clinical practice, patients with <10% blasts may also receive HMA if there is an anticipated prolonged interval between initial assessment and eventual allo-HCT, such as patients with a difficult donor search, or for hematologic improvement (expert opinion). However, the optimal cytoreduction regimen prior to transplant is not known [60]. HMA dose intensity pre-transplant is also dependent on the objectives of treatment (i.e., debulking vs. disease stability as a bridge to transplant), and treatment intensity needs to be weighed against potential complication risk that may ultimately preclude someone from undergoing a transplant.

Optimal time to transplant is a key determinant of outcomes. Delays to transplant are associated with higher risk of relapse [61], and optimal time to transplant impacts overall survival, whereas achieving composite complete response with HMAs prior to transplantation may not [62]. Thus, if a patient is fit and is undergoing cytoreduction for an imminent transplant, the standard initial dose of HMA (i.e., 5-day regimen of oral DEC-C) may be used (expert opinion). The reduced starting dose (i.e., 3-day regimen of oral DEC-C) may be considered to bridge patients to transplant in those with lower blast counts and those who have a prolonged wait for the procedure (e.g., to find a suitable donor), in order to minimize treatment-related risks that could impact transplant eligibility (expert opinion). Patients should generally not have transplant delayed to allow for additional HMA cycles or optimal response to HMA therapy.

## 3. Monitoring and Response Evaluation

**Figure 1:**
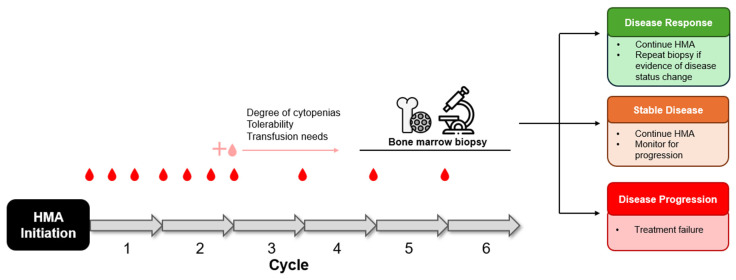
Real-world monitoring approach with HMAs. HMA: hypomethylating agent. 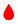 Represents complete blood count.

The monitoring approach for HMAs used in clinical practice is summarized in Figure 1. Following HMA initiation, complete blood cell counts should be performed at minimum prior to each cycle and as clinically indicated [3,5]. Clinicians leverage close monitoring (i.e., weekly) of complete blood cell counts for at least the first 2 cycles and then as indicated by degree of patient cytopenias, tolerability, and transfusion needs (expert opinion). Bone marrow biopsy for response assessment is standard to perform at the end of treatment cycles 4–6 [25] using the 2023 International Working Group criteria [63] (evidence-based). If the bone marrow biopsy shows attainment of disease response, the HMA is continued; additional biopsy is completed if there is evidence of disease status change, such as progression of cytopenias, to document disease recurrence vs. hypocellular marrow on treatment. If the bone marrow biopsy shows stable disease, the HMA may be continued until disease progression, even if clear hematologic improvement is not observed. Patients who have stable disease but do not achieve remission while on HMAs still experience survival benefits after HMA treatment [2,64]. If the bone marrow biopsy shows evidence of disease progression, the patient has treatment failure, and generally HMA is discontinued at this point.

## 4. Managing Myelosuppression

### 4.1. Timing of Dose Modification

Unless there are clinically significant complications (e.g., hospitalization, active infection), HMA dose modifications due to myelosuppression/cytopenias are typically not performed in the first ~4 cycles (oral DEC-C) or 4–6 cycles (azacitidine), as these cytopenias may still be attributed to disease activity [15] (expert opinion). If the patient is otherwise tolerating HMA therapy, clinicians continue with treatment until a minimum of 4–6 cycles are completed. Furthermore, although data are lacking, the clinical experience is that re-escalating the dose of HMA after dose reduction due to treatment-emergent AEs is rarely tolerated, and re-escalating dose for treatment failure is rarely successful in inducing disease response (expert opinion). Similarly, despite small case reports, switching HMA after failure is rarely successful and has little utility [65,66] (expert opinion).

### 4.2. Consideration of Thrombocytopenia

In HMA responders, the primary goals of treatment are to achieve transfusion independence and delay or prevent progression to AML. Asymptomatic cytopenias may be tolerated to achieve these goals. It is generally uncommon for patients who are red cell transfusion-independent to have critically low platelet counts; however, challenges can still arise in rare situations, such as in patients with very low baseline platelet counts. In such instances, prophylactic platelet transfusions are considered if the platelet count drops below 10 × 10^9^/L (expert opinion). For patients early in treatment (i.e., prior to cycle 4) who rely heavily on platelet transfusions, thrombocytopenia may be tolerated without adjusting the HMA dose to allow time for disease response when considering the risks of alloimmunization with frequent platelet transfusions (expert opinion). There is evidence that a doubling of the platelet count after the first cycle is predictive of HMA response [67]. If prior hematologic improvement of platelets has been achieved, but platelets remain suppressed by day 28, a treatment delay of 1–2 weeks may be necessary (expert opinion).

### 4.3. Consideration of Neutropenia

There are no strict thresholds for modifying HMA treatment due to neutropenia, as these decisions depend on the patient’s stage in treatment and their baseline values. For patients new to HMA therapy, there is greater impetus to tolerate low neutrophil counts (if not clinically significant) in order to continue treatment until 4–6 cycles to obtain a response. In general, clinicians in real-world practice may modify the HMA dose if the first 4 cycles have been completed and the absolute neutrophil count (ANC) drops below 0.5 × 10^9^/L, particularly in patients who did not present with severe neutropenia at diagnosis (expert opinion). In cases where the ANC is between 0.5 × 10^9^/L and 1.0 × 10^9^/L, supportive care measures (discussed later) or a dose delay may be considered (expert opinion). However, these decisions are often individualized based on the patient’s baseline and expected recovery. Gradual extension of the dosing cycle to every 5–6 weeks may also be considered for patients with mild neutropenia who are stable or in remission (expert opinion).

### 4.4. HMA Dose Modifications in Response to Myelosuppression

**Figure 2:**
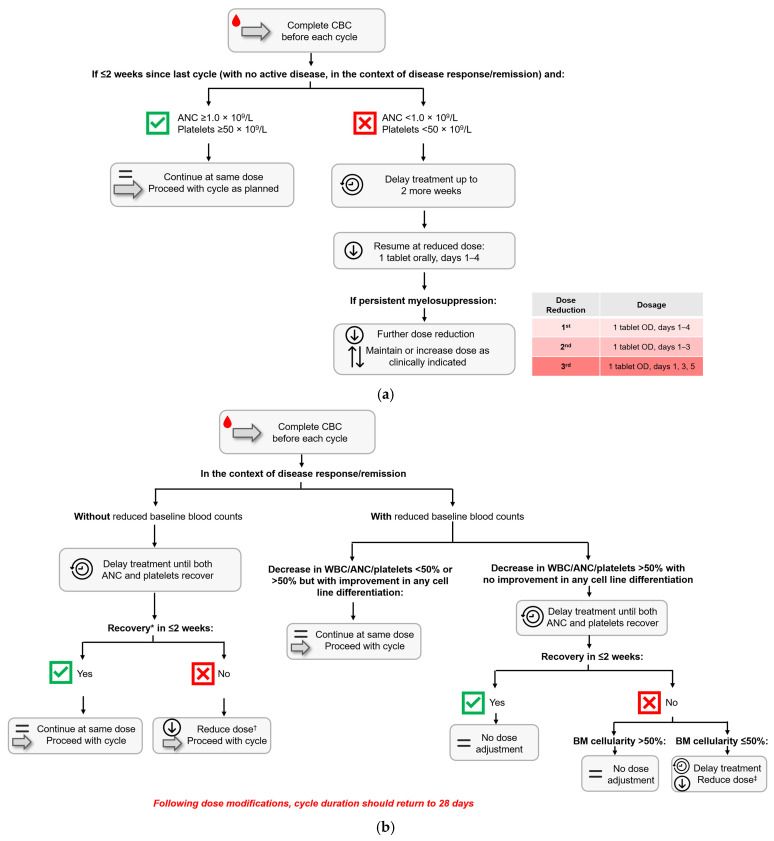
Dose modification of HMAs for hematologic AEs in the context of remission/response. (**a**) Oral DEC-C [5]; (**b**) Azacitidine [3]. * Recovery = counts ≥ nadir count + (0.5 × [baseline count − nadir count]); ^†^ If nadir ANC ≤1.0 × 10^9^/L and platelets ≤50 × 10^9^/L, reduce dose to 50%; ^‡^ If BM cellularity is 15–50% and recovery >21 days, reduce dose to 50%. If BM cellularity is <15% and recovery >21 days, reduce dose to 33%. ANC: absolute neutrophil count; BM: bone marrow; CBC: complete blood count; OD: once daily; WBC: white blood cell. 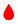 Represents complete blood count.

Health agency-recommended dose modification protocols are available for hematological AEs with both approved and available HMAs [3,5,41,42,49,50]. The Canadian product monograph-recommended approach to dose modification for myelosuppression, which should only be used after 4 cycles of treatment have been completed and in the context of disease response/remission, is summarized in Figure 2 (evidence-based)**.** Real-world approaches are supported by limited evidence, and the product monograph should remain the primary reference for dosing recommendations. In real-world clinical practice, the approach is to continue HMA monotherapy until disease response, then dose delay to allow for count recovery, and lengthen cycles or dose reduce to prevent prolonged cytopenias in subsequent cycles (expert opinion). Treatment with azacitidine should be delayed until both the platelet count and ANC recover. With azacitidine, if recovery occurs within 14 days, no dose adjustment is needed. If recovery does not occur within 14 days, the dose should be reduced by percentage depending on baseline blood counts and recovery as outlined in the product monograph. After dose modifications, the cycle duration should revert to 28 days [3] (evidence-based). Dose reductions of HMAs are generally avoided early in treatment but may be considered after patients have achieved response. This is generally earlier with DEC-C (median ~2 months) compared with azacitidine (median 3–4 months), based on anecdotal clinical experience (in the absence of comparative data) (expert opinion). Bone marrow biopsy to assess response is generally done after clinical improvement, which is by ~4 months with DEC-C and by at least 6 months with azacitidine.

Once the patient has been deemed to have achieved a response to HMA, oral DEC-C should be delayed for up to 2 additional weeks if the ANC is <1.0 × 10^9^/L and platelets are <50 × 10^9^/L within 2 weeks of the last treatment cycle. A reduced dosing regimen (i.e., 4 consecutive days) should be used upon resumption. If myelosuppression persists after dose reduction, further dosage reductions may be required. In subsequent cycles, the dosage should be maintained or increased as clinically indicated [5] (evidence-based). Canadian real-world experience showed 46.3% of patients receiving oral DEC-C required one or more dose reductions, whereas 53.7% had one or more dose delays [34]. This highlights that dose modifications—whether reductions or delays—are common in real-world use of oral DEC-C, reinforcing the need for proactive monitoring and flexible treatment planning to maintain efficacy while managing tolerability.

Both dose reduction (i.e., reducing the number of days of oral DEC-C or modifying the dose of azacitidine) and dose delays by extending the cycle interval are strategies that can help mitigate myelosuppression. There is little evidence to guide the preference of one strategy over the other; however, patients may prefer extending cycle lengths as it provides less time on treatment (expert opinion). HMAs do not induce minimal residual disease (MRD)-negative remissions; therefore, relapses after extended drug holidays are almost universal. There is limited evidence on the efficacy of HMA re-treatment in these cases (i.e., indefinite stop to treatment with a plan to restart if patient starts to progress) or re-treatment upon relapse, and thus it is not used in real-world practice at this time (expert opinion).

## 5. Supportive Care

Close monitoring for toxicity and frequent blood work are critical steps for proactive management of HMA-related AEs such as myelosuppression. Assessment of patients’ risk of infection is completed at diagnosis and investigations considered in at-risk patients, including viral disease screening (hepatitis B, hepatitis C, human immunodeficiency virus [HIV]), QuantiFERON^®^ testing for people at risk of latent tuberculosis infection, atypical infections in patients with extensive travel history, and *Strongyloides* serology in individuals from endemic regions (Caribbean, West and East Africa, and particularly Southeast Asia [68]) (expert opinion).

Treatment of gastrointestinal AEs (nausea, vomiting, diarrhea, constipation) is standard with use of both HMAs, including antiemetic prophylaxis (e.g., metoclopramide, ondansetron), as was used in clinical trials [2,4] (evidence-based). Skin reactions at the site of azacitidine injection are common and may be managed with application of evening primrose oil at the site and monitoring for infection [69] (expert opinion). Tranexamic acid is a useful adjunct to reduce mucosal bleeding in patients with platelets <10 × 10^9^/L or with platelets <30 × 10^9^/L and clinically relevant bleeding, although trial data specific to HMA-related myelosuppression are lacking [70,71] (expert opinion). Tranexamic acid is ‘relatively’ contraindicated in patients with hematuria [72]. There is no evidence at this time for use of thrombopoietin (TPO) receptor agonists in patients with higher-risk MDS, and these are generally avoided as some early trials demonstrated a possible increased risk of elevated blast count [73,74,75] (expert opinion).

Growth factors and antibiotic prophylaxis may be considered as supportive care for patients experiencing neutropenia during HMA treatment. Growth factors (granulocyte colony-stimulating factor [G-CSF]) were permitted for infections in patients with neutropenia in AZA-001, and short-term G-CSF was permitted for febrile neutropenia only in ASCERTAIN [4,76]. The use of G-CSF is variable. G-CSF may be used, most commonly as secondary prophylaxis, for patients with a history of infections or persistent severe neutropenia <0.5 × 10^9^/L with hypocellular marrow in remission on therapy, to avoid prolonged neutropenia (expert opinion). It may also be used if there are no excess blasts and repeat bone marrow demonstrates no disease progression (expert opinion). Use of G-CSF is avoided within 7 days prior to planned bone marrow aspirate to allow for accurate disease response assessment.

There is a considerable lack of data, especially robust, prospective studies, on the use of antimicrobials as primary prophylaxis for patients with MDS treated with HMA therapy. American Society of Clinical Oncology (ASCO)/Infectious Diseases Society of America (IDSA) guidelines recommend prophylactic antibiotic use for patients at high risk of febrile neutropenia and profound protracted neutropenia, although this is primarily informed by patients receiving intensive chemotherapy (i.e., 7 + 3) or those undergoing allo-HCT [77] (evidence-based). While antimicrobial prophylaxis (such as levofloxacin, valacyclovir, fluconazole, voriconazole) may be used in patients receiving HMAs with severe neutropenia (ANC <0.5 × 10^9^/L) or a history of prior infections, evidence to support its use is lacking and further studies are needed [78,79] (expert opinion). Concerns around drug resistance need to be balanced against the possible benefit of reducing infection risk, and this is an individualized decision. Ultimately, the use of growth factors and antimicrobial prophylaxis in patients with MDS receiving HMA treatment is highly variable across regions and centers in Canada. Clinicians should refer to institutional protocols for guidance on their use.

## 6. HMA Combination Therapy for MDS

Given the response rate to HMA monotherapy is only ~30–60%, there is an unmet need for more effective frontline interventions in patients with higher-risk MDS who are ineligible for transplant [2,4,80,81]. HMA + venetoclax combinations are currently being investigated in this setting for MDS; however, azacitidine + venetoclax failed to improve OS compared with azacitidine alone in the Phase III VERONA study [82]. Despite this, patients with excess blasts and certain genetic subgroups, like *ASXL1* and *RUNX1,* may selectively benefit in overall response if these agents are being used as bridges to transplant [83]. Oral DEC-C + venetoclax has shown early promise in patients with newly diagnosed AML who were ≥75 years of age or ineligible for intensive induction chemotherapy, where the all-oral regimen could help reduce treatment burden compared to current standard of care, parenteral azacitidine/decitabine + venetoclax [84]. Early data for oral DEC-C + venetoclax in MDS suggest good tolerability along with encouraging response and allo-HCT rates [85], but comparative data vs. monotherapy are not yet available. As a result, the precise role of HMA + venetoclax regimens in MDS continues to evolve and warrants further study. Furthermore, given high rates of cytopenias occurred with the combinations [82,85], strategies discussed herein (dose modifications and supportive care) will continue to be critical moving forward. Dose modification and supportive care strategies for combination therapy are even less well supported by evidence than those for HMA monotherapy and remain uncertain. In AML, AZA-VEN was associated with greater risk of prolonged cytopenias and resulting infection risk; thus, supportive care during the induction phase is critical to reduce infectious complications (expert opinion) [86]. Once remission is achieved, dose delays for marrow recovery and reduced duration of venetoclax (as opposed to lower dose) are strategies used to manage myelosuppression (expert opinion) [86]. The use of supportive G-CSF for patients in remission with neutropenia arising while on AZA + VEN has been adopted at many centers for patients with AML, with safety supported by retrospective and post hoc analyses, but its role in the context of combination therapy for patients with MDS remains unclear [87]. Future interventional and observational studies should focus on determining the optimal approaches for patients receiving HMAs, including dosing and supportive care with both monotherapy and combination, to address the lack of evidence on these critical aspects of MDS treatment.

## 7. Conclusions

The management of MDS with HMAs requires an individualized approach to mitigate myelosuppression. Close monitoring, dose modifications, and supportive care are essential to help prevent avoidable early discontinuation and allow patients to complete the necessary treatment cycles, thereby maximizing their chances for long-term benefit from HMA therapy. Continued research into optimizing HMA use and expanding therapeutic options remains critical to advancing the care of patients with MDS.

## Figures and Tables

**Table 1:** IPSS risk stratification system comparison.

	IPSS [12]	IPSS-R [13]	IPSS-M [9]
**Prognostic groups**	0 = Low 0.5–1.0 = INT-1 1.5–2.0 = INT-2 ≥2.5 = High	≤1.5 = Very low >1.5–3 = Low >3–4.5 = Intermediate >4.5–6 = High >6 = Very high	≤−1.5 = Very low >−1.5 to −0.5 = Low >−0.5 to 0 = Moderate low >0 to 0.5 = Moderate high >0.5 to 1.5 = High >1.5 = Very high
**Prognostic variables**	BM blasts: 0 = <5% 0.5 = 5–10% 1.5 = 11–20% 2.0 = 21–30% Cytogenetics: 0 = Good 0.5 = Intermediate 1.0 = Poor Cytopenias: Hemoglobin < 10 g/dL Platelets < 100,000/μL ANC < 1500/μL 0 = 0/1 cytopenias present 0.5 = 2/3 cytopenias present	BM blasts: 0 = ≤2% 1 = >2–<5% 2 = 5–10% 3 = >10% Cytogenetics: 0 = Very good 1 = Good 2 = Intermediate 3 = Poor 4 = Very poor Hemoglobin: 0 = ≥10 g/dL 1 = 8–<10 g/dL 1.5 = <8 g/dL Platelets: 0 = ≥100 × 10^9^/L 0.5 = 50–<100 × 10^9^/L 1 = <50 × 10^9^/L ANC: 0 = ≥0.8 × 10^9^/L 0.5 = <0.8 × 10^9^/L	Somatic mutations: 31 genes BM blasts: Continuous Cytogenetics: Integrated with molecular findings Hemoglobin: Continuous Platelets: Continuous

ANC: absolute neutrophil count; BM: bone marrow; INT: intermediate; IPSS: International Prognostic Scoring System; IPSS-M: Molecular International Prognostic Scoring System; IPSS-R: International Prognostic Scoring System-Revised.

**Table 2:** Response to HMAs in pivotal clinical trials for MDS.

	Azacitidine (AZA-001) [2]	Oral DEC-C (ASCERTAIN) [4]
	Months	Months
**Median time to first response**	1.8 (range 0.9–14.7) [28]	1.9 (IQR 1.2–3.8)
**Median time to best response**	From first response: 3.2 (95% CI 2.8–5.5) in patients who ultimately achieved a CR [28]	From treatment start: 3.3 (IQR 1.8–5.3)
**Median duration of response**	13.6 (IQR 5.9–26.4, 95% CI 10.1–16.3) (hematological response)	12.2 (95% CI 9.5–14.4) (best response); 14.1 (95% CI 11.7–18.7) (CR)
	**Cycles**	**Cycles**
**Median number of cycles**	9 (IQR 4–15)	9 (IQR 4–17)

Data presented are from separate clinical trials and should not be interpreted as head-to-head efficacy or safety comparisons. CI: confidence interval; CR: complete response; DEC-C: decitabine-cedazuridine; HMA: hypomethylating agent; IQR: interquartile range; MDS: myelodysplastic syndrome.

**Table 3:** Myelosuppression and infection incidence in HMA pivotal clinical trials for MDS.

% of Participants	Azacitidine (*n* = 179) (AZA-001) [2]	Oral DEC-C (*n* = 133) (ASCERTAIN) [4]
	Grade ≥ 3	Grade ≥ 3
**Neutropenia**	91	57
**Thrombocytopenia**	85	61
**Anemia**	57	50
**Febrile neutropenia**	12.6 * [3]	32
**URTI**	1.7 * [3]	1 ^†^ [5]
**UTI**	1.7 * [3]	Not reported [5]
**Pneumonia**	SAE ^‡^: 1.7 [3]	15 ^†^ [5]
**Sepsis**	SAE ^‡^: 1.7 [3]	11 ^†^ [5]
**Cellulitis**	Not reported [3]	5 ^†^ [5]

Data presented are from separate clinical trials and should not be interpreted as head-to-head efficacy or safety comparisons. * *n* = 175; ^†^ Pooled safety population (all cycles), *n* = 208; ^‡^ Reported as a serious adverse event, not specifically Grade ≥3; DEC-C: decitabine-cedazuridine; HMA: hypomethylating agent; MDS: myelodysplastic syndrome; SAE: serious adverse event; URTI: upper respiratory tract infection; UTI: urinary tract infection.

## Data Availability

No new data were created or analyzed in this study. Data sharing is not applicable to this article.
